# A Mendelian randomization study on causal effects of 25(OH) vitamin D levels on diabetic nephropathy

**DOI:** 10.1186/s12882-023-03186-2

**Published:** 2023-06-27

**Authors:** Mingjie He, Ting Yang, Ping Zhou, Peiyan Bu, Xionghui Yang, Yan Zou, Aimin Zhong

**Affiliations:** 1 Jiangxi Provincial Key Laboratory of Nephrology, Jiangxi Provincial People’s Hospital, The First Affiliated Hospital of Nanchang Medical College, Nanchang, China; 2 Medical College of Nanchang University, Nanchang University, 330006 Nanchang City, China

**Keywords:** Vitamin D, Diabetic nephropathy, Mendelian randomization

## Abstract

**Background:**

Vitamin D supplementation is associated with a lower incidence of diabetic nephropathy (DN); however, whether this association is causative is uncertain.

**Methods:**

We used two-sample Mendelian randomization to examine the causal influence of vitamin D on diabetic nephropathy in 7,751 individuals with type I diabetes-related nephropathy (T1DN) and 9,933 individuals with type II diabetes-related nephropathy (T2DN). Meanwhile, we repeated some previous studies on the influence of KIM-1 (kidney injury molecule 1) and body mass index (BMI) on DN. Additionally, to test the validity of the instruments variable for vitamin D, we conducted two negative controls Mendelian randomization (MR) on breast and prostate cancer, and a positive control MR on multiple sclerosis.

**Results:**

Results of the MR analysis showed that there was no causal association between 25(OH)D with the early/later stage of T1DN (early: OR = 0.903, 95%CI: 0.229 to 3.555; later: OR = 1.213, 95%CI: 0.367 to 4.010) and T2DN (early: OR = 0.588, 95%CI: 0.182 to 1.904; later: OR = 0.904, 95%CI: 0.376 to 2.173), nor with the kidney function of patients with diabetes mellitus: eGFRcyea (creatinine-based estimated GFR) (Beta = 0.007, 95%CI: -0.355 to 0.369)) or UACR (urinary albumin creatinine ratio) (Beta = 0.186, 95%CI: -0.961 to 1.333)).

**Conclusions:**

We found no evidence that Vitamin D was causally associated with DN or kidney function in diabetic patients.

**Supplementary Information:**

The online version contains supplementary material available at 10.1186/s12882-023-03186-2.

## Introduction

Diabetic nephropathy (DN) is the leading cause of end-stage renal disease (ESRD) and is associated with a high risk of cardiovascular disease. Proteinuria, hypertension, and gradual declines in kidney function are the clinical manifestations [1]. At present, the clinical treatment of DN can roughly be divided into four major areas: cardiovascular risk reduction, glycemic control, BP control, and inhibition of the renin-angiotensin system (RAS) [2].

Vitamin D can potentially protect against DN [3, 4]. It is widely accepted that vitamin D improves calcium levels, decreasing the risk of CKD-MBD (chronic kidney disease-mineral and bone disorder) [5, 6]. Emerging evidence depicts that vitamin D may improve glucose metabolism, lower RAS activation, and inhibit fibrosis [7, 8]. Vitamin D produces a therapeutic impact only if it is converted to its active form through metabolism. The main circulating form of the vitamin, 25(OH)D, involves many genes, such as *CYP2R1*, *AMDHD1*, *NADSYN1-DHCR7*, and *CYP24A1* [9]. Some reports have revealed that 25(OH)D can attenuate renin expression, suppressing the RAS system, a key contributor to DN [4, 10].

Vitamin D deficiency is widely believed to be associated with the development of diabetic nephropathy and type I and type II diabetes mellitus [8, 11]. A multicenter randomized controlled trial that measured the vitamin D levels in 103 patients at baseline, 4, and 12 months found that 25(OH)D deficiency accelerated the progression of chronic kidney disease (CKD) in patients with T2DN [10]. In contrast, a meta-analysis of nine random control trials (RCTs) involving 828 patients demonstrated that vitamin D might have a non-significant effect on slowing the progression of diabetic nephropathy [12]. Another larger meta-analysis, which included 20 RCTs representing 1,464 patients with DN, found that vitamin D can reduce the levels of UACR and 24-h urine protein but not the eGFR [12, 13]. A clinical trial including 240 patients with type 2 diabetes mellitus found that 25(OH)D may not be associated with different stages of renal failure, while it could affect the level of microalbuminuria [13, 14]. Thus, the causal relationship between vitamin D and DN remains to be proven.

Observational studies are frequently susceptible to confounding factors. Mendelian randomization (MR), a method used for causal inference in epidemiology, limits bias due to confounding and reverse causation which is common in observational studies. MR analyzes the causal relationship between exposure and outcome using unconfounded instrumental variables. The instrumental variables include single nucleotide polymorphisms (SNP), which are strongly associated with exposure [15]. Furthermore, MR can reduce the bias caused by reverse causation [16].

**Fig. 1:**
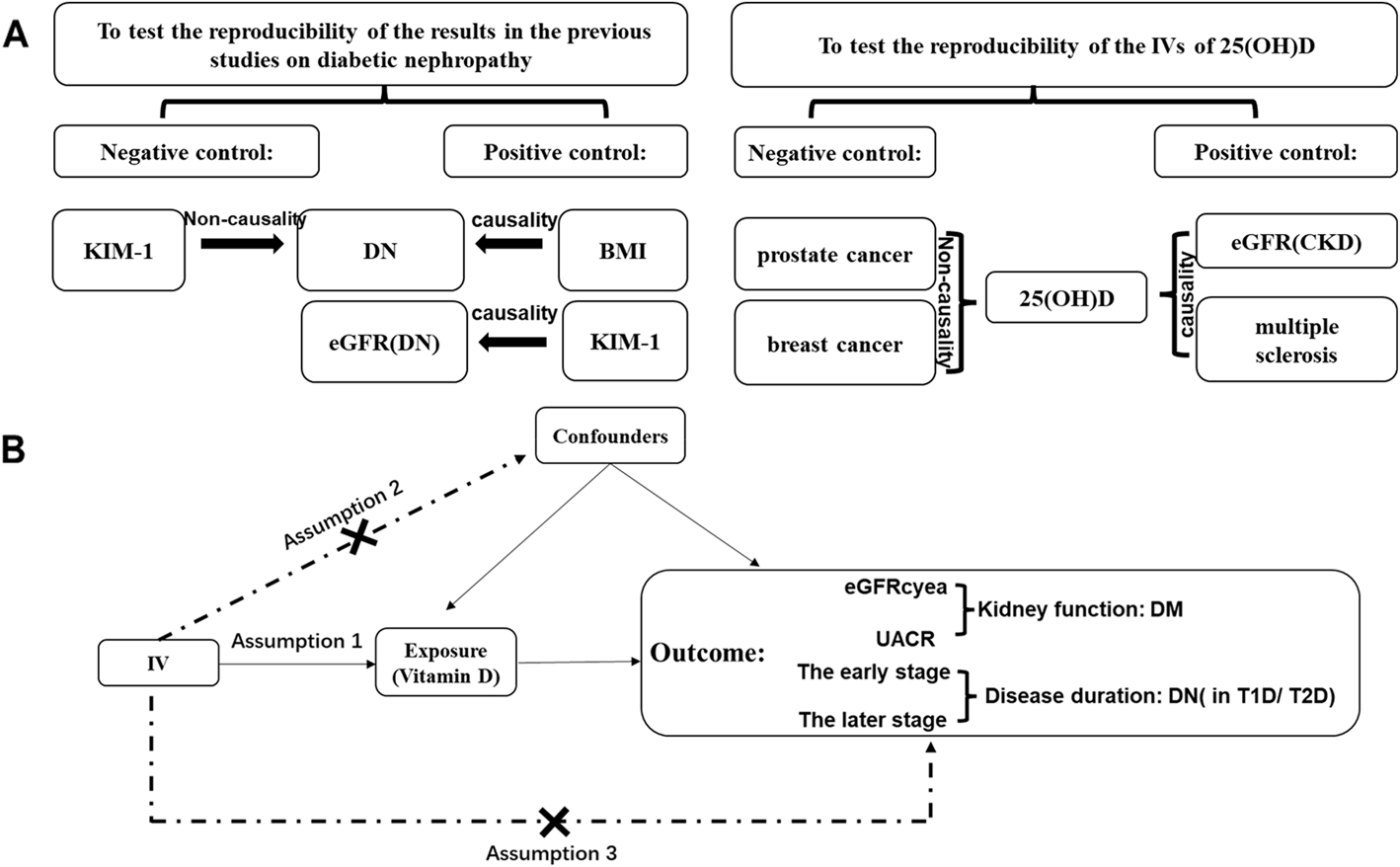
**A** Design of control groups. **B** Basic assumptions of mendelian randomization and main design of this study. the IV, instrumental variable. eGFRcyea, creatinine-based estimated glomerular filtration rate. UACR, urine Albumin-to-Creatinine Ratio. DM, diabetes mellitus. DN, diabetic nephropathy. T1D, type 1 diabetes. T2D, type 2 diabetes

In this study, we investigated the relationship between the levels of circulating 25(OH)D with the disease duration of DN, eGFR, and UACR in diabetes mellitus, using the MR approach. To verify the validity of the IVs and the reproducibility of the previous study, MR studies were conducted as numerous positive controls (PC) and negative controls (NC), shown in Fig. 1. The positive controls indicate that there is a causal relationship between outcome and exposure, while the NC revealed that there is no causal relationship. First, because the IVs of vitamin D that we utilized needed to be tested, we established two positive control groups, including patients with multiple sclerosis [17] and those with CKD [18], as well as two negative control groups, including those with prostate cancer and breast cancer [19]. Second, we wanted to test the reproducibility of the results in the previous studies, which showed that BMI was connected with DN and KIM-1 was associated with the kidney function of DN independent of the disease duration [20, 21].

## Methods

### Two-sample MR design

Two-sample Mendelian randomization (TSMR) is based on three basic assumptions: (1) SNPs are associated with the exposure, (2) no association between SNPs with confounders of the exposure-outcome association, and (3) SNPs are associated with the outcome only through exposure (Fig. 1). At the same time, these SNPs associated with 25(OH)D levels were selected based on *p* < 5 × 10^–8^ and minor allele frequency > 0.01. Furthermore, we calculated the F statistics of SNPs to investigate the presence of weak instrument bias [22].

### GWAS sources for exposures

We extracted instrumental variables (IVs) of Vitamin D from a genome-wide association study (GWAS) analysis with 79,366 European-ancestry individuals, displayed in ST-1 [23]. There are four SNPs (involving genes having a direct role in vitamin D synthesis and metabolism) that explain 2.84% of the increase in 25OHD levels: rs10741657 (*CYP2R1*), rs10745742 (*AMDHD1*), rs12785878 (*NADSYN1-DHCR7*), rs17216707(*CYP24A1*). The total F statistic for these four SNPS was 579.93. Because rs17216707(*CYP24A1*) is associated with kidney function (ST-2), we removed this SNP. Furthermore, to expand the power of vitamin D IVs, we extracted 138 SNPs from a larger GWAS study by Manousaki et al., including 443,734 European individuals (ST-8) [9]. The proportion of the variance of Vitamin D explained by 138 SNPs was 0.0834 and the total F statistic of 138 SNPs was 294.77. BMI and KIM-1 were used as two positive groups. The IVs of BMI were the 10 lead SNPs reported in the largest European GWAS of obesity published by *Speliotes* et al., shown in ST-3 [24]. The significant association between IVs of BMI with phenotype was displayed in the ST-4. The IV of KIM-1 (Kidney Injury Molecule-1) was rs1039438 (Beta = -0.5, *p* = 7.81E-38) extracted from the study by *Per-Henrik Groop*, shown in ST-5 [20].

### GWAS sources for outcomes

This MR analysis aims to clarify the causal relationship between serum 25(OH)D and DN. Therefore, we used two types of data: the GWAS summary statistics of eGFR and UACR with diabetes and the GWAS summary data on the different stages of DN.

The GWAS summary data on eGFR in patients with CKD was obtained from a GWAS analysis based on 133,814 European-ancestry individuals [25]. The eGFR was defined by the four-variable Modification of Diet in the Renal Disease Study Equation. The UACR was calculated as urinary albumin/urinary creatinine (mg/g). The values of eGFR and UACR are obtained by log () transformation.

The GWAS summary statistic of eGFR and UACR in patients with diabetes mellitus was derived from two GWAS studies that included 133,814 and 51,886 individuals of European ancestry, respectively [25, 26]. Diabetes mellitus was defined as fasting glucose ≥ 126 mg/dl, pharmacologic treatment for diabetes, or self-report.

The GWAS summary statistics of the early/later DN in patients with type I diabetes comprises 3,399 and 4,352 European [26]. The definition of type I diabetes is diagnosed by their attending physician, with age at diabetes onset < 40 years and insulin treatment initiated within 1 year of diagnosis. The early DN was characterized by “at least 2 out of 3 consecutive measurements with AER ≥ 20 AND < 200 mg/min” or “AER ≥ 30 AND, < 300 mg/24 h” or “ACR ≥ 2.5/3.5 AND, < 25/35 mg/mmol.” The term “later type I diabetes-related nephropathy” was used to describe patients who were on dialysis, had received a kidney transplant, or had an eGFR ≤ 15 mL/min per 1.73 m^2^. The statistics were adjusted for sex, diabetes duration, and age at diabetes onset.

The GWAS summary statistics of the early/later DN in type II diabetes were derived from an analysis of 4,805 and 5,128 European individuals [27]. The “early DKD” phenotype identifies variants that contribute to the early dysfunction of the glomerular barrier. The “late DKD” phenotype identifies variants that contribute to severe glomerular barrier dysfunction. The statistics were adjusted for sex, diabetes duration, and age at diabetes onset.

The GWAS summary statistic of prostate cancer was obtained from 2,495 cases and 334,644 controls at Neale Lab (http://www.Nealelab.is/UK-biobank) using Hail (https://hail.is/), with adjustment of the first 20 principal components, sex, age, age squared, the interaction between sex and age, and interaction between sex and age squared.

The GWAS summary statistic of breast cancer from Neale Lab included 25,865 cases and 283,784 controls, with adjustments for sex, age, age squared, the interaction between sex and age, and the interaction between sex and age squared.

The GWAS summary statistic for multiple sclerosis includes 47,429 cases and 68,374 controls in the International Multiple Sclerosis Genetics Consortium [28].

### Statistical analysis

We used the inverse variance-weighted (IVW) method to estimate the effect of 25(OH)D on DN. Weighted-median was used as a supplementary method to IVW, as depicted in ST-6. For the KMI with one IV, only Wald ratios were presented. Simultaneously, we chose the random effects model IVW according to the result of heterogeneity [29]. Heterogeneity is assessed by Cochrane’s Q value. The horizontal pleiotropy of SNPs was evaluated through the MR-Egger intercept [10] and MR-PRESSO methods [30]. Specifically, the outlier test corrects for horizontal pleiotropy by removing outliers. The MR Steiger directionality test was utilized to assume whether the direction of causality is correct when there was a causal relationship between exposure and outcome in MR analysis [31]. Power was estimated using an online tool, mRnd (https://cnsgenomics.shinyapps.io/mRnd/) (ST-10) [32]. The F-statistic can be calculated using the following formula: F = (R^2^/k)/ ([1 − R^2^]/ [n − k − 1]), where R^2^ is the proportion of the variance of vitamin D explained by all SNPs, k is the number of SNP-instruments used in the model and n is the GWAS sample size. R^2^ is estimated by $2\times \beta^2\times EAF\times (1-EAF)$, where β is the effect estimate and EAF is the effect allele frequency of the allele [33, 34].

Two sample Mendelian randomization analysis was performed using the R package “TwoSampleMR” [35]. The MR-PRESSO was conducted using the R package “MRPRESSO” [30]. The association between SNPs and phenotype was analyzed by “PhenoScanner V2” [36]. All statistical analyses were performed using R software version 4.1.2 (https://www.r-project.org/).

## Results

### Assessing the validity of the instrumental variables

To test the reproducibility of the results in previous studies on DN and the reliability of the IVs of 25(OH)D, we designed the positive control and negative control study (Table 1 and ST-6). We replicated the previous result that 25(OH)D levels was inversely associated with the risk of multiple sclerosis (OR = 0.327, 95%CI: 0.151 to 0.709) [17] and reduced eGFRcyea in patients with CKD disease (Beta = -0.053, 95%CI: -0.09 to -0.016), whereas serum 25(OH)D was not significantly associated with the increasing risk of breast cancer (OR = 1.027, 95%CI: 0.996 to 1.06) and prostate cancer (OR = 1.008, 95%CI: 0.999 to 1.017) [19]. Horizontal pleiotropy was not detected in this analysis. Heterogeneity could be found in the eGFRcyea, which we used the random effects model IVW to correct it (ST-6). Moreover, we found that the causal effect was true between 25(OH)D and both Multiple sclerosis and CKD (eGFRcyea) by using Steiger-test (ST-7).

**Table 1:** The IVW values for MR. IVs in the MR study means that IVs that actually participate in the MR study, after harmonising the information from exposure and outcome

Diseases	IVs in the MR study	Factors	*p*.value	95%LCI	OR/Beta	95%UCI
**DN**	**3**	**25(OH)D**	**0.987**	**0.269**	**1.011**	**3.793**
**T1DN(early)**	**3**	**25(OH)D**	**0.726**	**0.030**	**0.587**	**11.458**
**T1DN(later)**	**3**	**25(OH)D**	**0.752**	**0.114**	**1.517**	**20.208**
**T2DN(early)**	**3**	**25(OH)D**	**0.109**	**0.001**	**0.039**	**2.075**
**T2DN(later)**	**3**	**25(OH)D**	**0.435**	**0.389**	**1.870**	**8.990**
**Prostate cancer**	**3**	**25(OH)D**	**0.087**	**0.999**	**1.008**	**1.017**
**Breast cancer**	**3**	**25(OH)D**	**0.092**	**0.996**	**1.027**	**1.060**
**Multiple sclerosis**	**2**	**25(OH)D**	**0.005**	**0.151**	**0.327**	**0.709**
**CKD(eGFRcyea)**	**3**	**25(OH)D**	**0.005**	**-0.090**	**-0.053**	**-0.016**
**DM(eGFRcyea)**	**3**	**25(OH)D**	**0.971**	**-0.355**	**0.007**	**0.369**
**DM(UACR)**	**3**	**25(OH)D**	**0.751**	**-0.961**	**0.186**	**1.333**
**DN**	**9**	**BMI**	**0.034**	**1.007**	**1.102**	**1.206**
**T1DN(early)**	**9**	**BMI**	**0.700**	**0.880**	**0.979**	**1.090**
**T1DN(later)**	**9**	**BMI**	**0.001**	**1.117**	**1.322**	**1.564**
**T2DN(early)**	**8**	**BMI**	**0.661**	**0.832**	**1.054**	**1.336**
**T2DN(later)**	**8**	**BMI**	**0.142**	**0.946**	**1.180**	**1.473**
**CKD(eGFRcyea)**	**8**	**BMI**	**0.131**	**-0.010**	**-0.004**	**0.001**
**DM(eGFRcyea)**	**8**	**BMI**	**0.730**	**-0.017**	**0.004**	**0.024**
**DM(UACR)**	**8**	**BMI**	**0.011**	**0.034**	**0.149**	**0.264**
**CKD(eGFRcyea)**	**1**	**KIM-1**	**0.016**	**-0.009**	**-0.005**	**-0.001**
**DM(eGFRcyea)**	**1**	**KIM-1**	**0.043**	**-0.032**	**-0.016**	**-0.001**
**DM(UACR)**	**1**	**KIM-1**	**0.652**	**-0.150**	**-0.028**	**0.094**
**DN**	**1**	**KIM-1**	**0.125**	**0.815**	**0.914**	**1.025**
**D1N(early)**	**1**	**KIM-1**	**0.374**	**0.753**	**0.915**	**1.113**
**D1N(later)**	**1**	**KIM-1**	**0.123**	**0.950**	**1.209**	**1.539**
**D2N(early)**	**1**	**KIM-1**	**0.227**	**0.911**	**1.160**	**1.477**
**D2N(later)**	**1**	**KIM-1**	**0.352**	**0.847**	**1.162**	**1.593**

*95%LCI* The lower limit of 95% CI, *95%UCI* The upper limit of 95% CI, *T1DN* Type 1 diabetic neuropathy, *T2DN* Type 2 diabetic neuropathy, *DM* Diabetic mellitus, *DN* Diabetic nephropathy, *CKD* Chronic kidney disease

Previous studies have demonstrated that risk factors, such as KIM-1, can affect renal function independent of the disease duration in patients with DN [20], while BMI can directly promote the progression of DN [21]. Our study found that BMI can increase the risk of DN (OR = 1.102, 95%CI: 1.007 to 1.206, p_Steiger_test_ < 0.0001), and a further novel finding is that BMI can only promote T1DN at the later stage (OR = 1.322, 95%CI: 1.117 to 1.564, p_Steiger_test_ < 0.0001). For the kidney function of individuals with diabetes mellitus, BMI was not negatively correlated with eGFRcrea (Beta = -0.004, 95%CI: -0.017 to 0.024) as previously reported, but was associated with UACR (Beta = 0.149, 95%CI: 0.034 to 0.264, p_Steiger_test_ < 0.0001). Similarly, our MR analysis depicted that KIM-1 affects kidney function in individuals with diabetes mellitus through eGFRcrea (Beta = -0.016, 95%CI: -0.032 to -0.001, p_Steiger_test_ < 0.0001) independently of disease duration (DN: OR = 0.914, 95%CI: 95%CI: 0.815 to 1.025).

Together, the present findings confirm that the IVs are reliable and the result in the previous studies is reproducible (ST-7).

### Assessing the association between vitamin D and DN

To elaborate on the effects of vitamin D on DN, we focused on the progression of diabetic nephropathy and the kidney function of individuals with diabetes (Table 1 and ST-6,9). Horizontal pleiotropy and heterogeneity were not detected in these analyses (ST-6,9).

The results of the MR analysis suggested that serum 25(OH)D level appears to promote the risk of DN, but there was no statistical association (OR = 1.011, 95%CI: 0.269 to 3.793). The statistical power for this result was 0.05. Nevertheless, the disease’s duration of DN or the type of DN may disturb our MR analysis. Hence, we used the GWAS statistic of DN in the early and later stages. We also could not find a statistical difference to support the causal relationship between 25(OH)D and DN(T1DN (later): OR = 1.517 (95%CI: 0.114 to 20.208); T1DN(early): OR = 0.587 (95%CI: 0.03 to 11.458); T2DN(early): OR = 0.039 (95%CI: 0.001 to 2.075); T2DN(later): OR = 1.87 (95%CI: 0.389 to 8.99)). Meanwhile, our MR study found that 25(OH)D could not affect kidney function in patients with diabetes, eGFRcyea (Beta = 0.007, 95%CI: -0.355 to 0.369) or UACR (Beta = 0.186, 95%CI: -0.961 to 1.333). The power analysis showed that those results had more than 80% power, except for eGFRcyea in patients with diabetes, depicted in ST-10.

However, in the above MR results, the confidence interval was too wide to interpret the direction of OR. Thus, we used the IVs of vitamin D from another study [9]. We found that the confidence interval calculated with 138 SNPs was narrower than with 3 SNPs, which was more convincing, shown in ST-10. There was also no evidence to support the association between vitamin D and DN (OR = 1.755, 95%CI: 0.802 to 3.841), shown in ST-9. The statistical power for this result was 100%. But the OR results demonstrated that 25(OH)D may increase the risk in the later stage of T1DN (OR = 1.213, 95%CI: 0.367 to 4.010), while decreasing the risk in the early stage of T1DN (OR = 0.903, 95%CI: 0.229 to 3.555), and T2DN (early: OR = 0.588, 95%CI: 0.182 to 1.904; later: OR = 0.904, 95%CI: 0.376 to 2.173). The power analysis showed that those results had less than 80% power, except for the early stage of T2DN, depicted in ST-10.

## Discussion and conclusions

Vitamin D is a pleiotropic lipid-soluble vitamin that not only regulates calcium and phosphorus metabolism but also has immune-boosting properties [37]. Although vitamin D has been used in patients with DN, the association between vitamin D and DN remains contentious, and our MR analysis indicates that 25(OH)D does not directly affect the clinical course of early-stage T1DN and the early/later-stage T2DN. Furthermore, some reports have revealed that 25(OH)D can protect the kidney function of patients with CKD, which is verified by our results [10]. For patients with diabetes mellitus, our analysis suggests a favorable trend concerning the effect of 25(OH)D on eGFR and UACR without significant differences.

We also discuss the effect of KIM-1 and BMI on DN progression and kidney function in patients with CKD or diabetes mellitus. As previously reported, our MR results support the notion KIM-1 is not an independent predictor of the progression of T1DN or T2DN. However, its levels may have a causal link with eGFR. BMI, an indicator of obesity, has been reported to be causally associated with an increased risk of DN [21]. Our MR provides evidence to suggest that BMI is only causally associated with the later stage of T1DN, however, further studies are needed to confirm this.

However, our MR study has many limitations. The major assumption in MR analysis is that genetic variants affect DN only through vitamin D concentrations. Although we used the MR-Egger intercept to control pleiotropy, the impact of unknown functions on genetic variants may influence DN independently. At the same time, our MR study can only test the linear effect of circulating vitamin D concentrations in the general population. Thus, more individual data are required for nonlinear MR, implying potential nonlinear relationships between vitamin D and DN. Although statistical power was enough in the MR results of the early and later stages of DN by using 3 SNPs, the confidence interval was too wide to interpret the direction of the effect. At the same time, the confidence interval was narrowed by using 138 SNPs, but the statistical power was less than 80%. Thus, a larger sample size of the early and later stages of DN is needed to contribute to revealing the relationship between Vitamin D and the progression of DN.

In conclusion, our result indicated that 25(OH)D might reduce the risk of DN, either for T1DN or for T2DN in the early stage. To confirm this, additional individual data and a larger sample size are required. In addition, to clarify the mechanisms by which vitamin D protects patients with DN, our further work will harness experimental data to explore the function of 25(OH)D in DN.

## Supplementary Information

**Additional file 1. **

## Data Availability

Type 1 Diabetes Knowledge Portal: (https://t1d.hugeamp.org/), under “SUMMIT Diabetic Kidney Disease GWAS: subjects with T1D, Europeans” datasets. Type 2 Diabetes Knowledge Portal: (https://t2d.hugeamp.org/), under “SUMMIT Diabetic Kidney Disease GWAS: subjects with T2D, Europeans” datasets. UKB: (http://www.nealelab.is/uk-biobank). CKDGen: (https://ckdgen.imbi.uni-freiburg.de/).

## Abbreviations

DN: Diabetic nephropathy
T1DN: Type I diabetic-related nephropathy
T2DN: Type II diabetic-related nephropathy

## References

[1] Oshima M, Shimizu M, Yamanouchi M, Toyama T, Hara A, Furuichi K (2021). Trajectories of kidney function in diabetes: a clinicopathological update. Nat Rev Nephrol.

[2] Umanath K, Lewis JB (2018). Update on diabetic nephropathy: core curriculum 2018. Am J Kidney Dis.

[3] Esfandiari A, Pourghassem Gargari B, Noshad H, Sarbakhsh P, Mobasseri M, Barzegari M (2019). The effects of vitamin D(3) supplementation on some metabolic and inflammatory markers in diabetic nephropathy patients with marginal status of vitamin D: a randomized double blind placebo controlled clinical trial. Diabetes Metab Syndr.

[4] Hu X, Liu W, Yan Y, Liu H, Huang Q, Xiao Y (2019). Vitamin D protects against diabetic nephropathy: evidence-based effectiveness and mechanism. Eur J Pharmacol.

[5] Plum LA, Zella JB (2012). Vitamin D compounds and diabetic nephropathy. Arch Biochem Biophys.

[6] Rastogi A, Bhatt N, Rossetti S, Beto J (2021). Management of hyperphosphatemia in end-stage renal disease: a new paradigm. J Ren Nutr.

[7] Lei M, Liu Z, Guo J (2020). The emerging role of vitamin D and vitamin D receptor in diabetic nephropathy. Biomed Res Int.

[8] Yang L, Wu L, Fan Y, Ma J (2017). Vitamin D receptor gene polymorphisms in association with diabetic nephropathy: a systematic review and meta-analysis. BMC Med Genet.

[9] Manousaki D, Mitchell R, Dudding T, Haworth S, Harroud A, Forgetta V (2020). Genome-wide association study for vitamin D levels reveals 69 independent loci. Am J Hum Genet.

[10] Fernandez-Juarez G, Luno J, Barrio V, de Vinuesa SG, Praga M, Goicoechea M (2013). 25 (OH) vitamin D levels and renal disease progression in patients with type 2 diabetic nephropathy and blockade of the renin-angiotensin system. Clin J Am Soc Nephrol.

[11] Mitri J, Pittas AG (2014). Vitamin D and diabetes. Endocrinol Metab Clin North Am.

[12] Gupta S, Goyal P, Feinn RS, Mattana J (2019). Role of vitamin D and its analogues in diabetic nephropathy: a meta-analysis. Am J Med Sci.

[13] Wang Y, Yang S, Zhou Q, Zhang H, Yi B (2019). Effects of vitamin D supplementation on renal function, inflammation and glycemic control in patients with diabetic nephropathy: a systematic review and meta-analysis. Kidney Blood Press Res.

[14] Ucak S, Sevim E, Ersoy D, Sivritepe R, Basat O, Atay S (2019). Evaluation of the relationship between microalbuminuria and 25-(OH) vitamin D levels in patients with type 2 diabetes mellitus. Aging Male.

[15] Sekula P, Del Greco MF, Pattaro C, Köttgen A (2016). Mendelian randomization as an approach to assess causality using observational data. J Am Soc Nephrol.

[16] Davey Smith G, Hemani G (2014). Mendelian randomization: genetic anchors for causal inference in epidemiological studies. Hum Mol Genet.

[17] Mokry LE, Ross S, Ahmad OS, Forgetta V, Smith GD, Goltzman D (2015). Vitamin D and risk of multiple sclerosis: a Mendelian randomization study. PLoS Med.

[18] Teumer A, Gambaro G, Corre T, Bochud M, Vollenweider P, Guessous I (2018). Negative effect of vitamin D on kidney function: a Mendelian randomization study. Nephrol Dial Transplant.

[19] Jiang X, Dimou NL, Al-Dabhani K, Lewis SJ, Martin RM, Haycock PC (2019). Circulating vitamin D concentrations and risk of breast and prostate cancer: a Mendelian randomization study. Int J Epidemiol.

[20] Panduru NM, Sandholm N, Forsblom C, Saraheimo M, Dahlstrom EH, Thorn LM (2015). Kidney injury molecule-1 and the loss of kidney function in diabetic nephropathy: a likely causal link in patients with type 1 diabetes. Diabetes Care.

[21] Todd JN, Dahlstrom EH, Salem RM, Sandholm N, Forsblom C, FinnDiane Study G (2015). Genetic evidence for a causal role of obesity in diabetic kidney disease. Diabetes..

[22] Burgess S, Thompson SG, Collaboration CCG (2011). Avoiding bias from weak instruments in Mendelian randomization studies. Int J Epidemiol.

[23] Jiang X, O'Reilly PF, Aschard H, Hsu YH, Richards JB, Dupuis J (2018). Genome-wide association study in 79,366 European-ancestry individuals informs the genetic architecture of 25-hydroxyvitamin D levels. Nat Commun.

[24] Speliotes EK, Willer CJ, Berndt SI, Monda KL, Thorleifsson G, Jackson AU (2010). Association analyses of 249,796 individuals reveal 18 new loci associated with body mass index. Nat Genet.

[25] Pattaro C, Teumer A, Gorski M, Chu AY, Li M, Mijatovic V (2016). Genetic associations at 53 loci highlight cell types and biological pathways relevant for kidney function. Nat Commun.

[26] Teumer A, Tin A, Sorice R, Gorski M, Yeo NC, Chu AY (2016). Genome-wide association studies identify genetic Loci Associated With Albuminuria in Diabetes. Diabetes.

[27] van Zuydam NR, Ahlqvist E, Sandholm N, Deshmukh H, Rayner NW, Abdalla M (2018). A Genome-wide association study of diabetic kidney disease in subjects with type 2 diabetes. Diabetes.

[28] International Multiple Sclerosis Genetics C (2019). Multiple sclerosis genomic map implicates peripheral immune cells and microglia in susceptibility. Science.

[29] Burgess S, Thompson SG (2017). Interpreting findings from Mendelian randomization using the MR-Egger method. Eur J Epidemiol.

[30] Verbanck M, Chen CY, Neale B, Do R (2018). Detection of widespread horizontal pleiotropy in causal relationships inferred from Mendelian randomization between complex traits and diseases. Nat Genet.

[31] Hemani G, Tilling K, Davey SG (2017). Orienting the causal relationship between imprecisely measured traits using GWAS summary data. PLoS Genet.

[32] Brion MJ, Shakhbazov K, Visscher PM (2013). Calculating statistical power in Mendelian randomization studies. Int J Epidemiol.

[33] Palmer TM, Lawlor DA, Harbord RM, Sheehan NA, Tobias JH, Timpson NJ (2012). Using multiple genetic variants as instrumental variables for modifiable risk factors. Stat Methods Med Res.

[34] Park JH, Wacholder S, Gail MH, Peters U, Jacobs KB, Chanock SJ (2010). Estimation of effect size distribution from genome-wide association studies and implications for future discoveries. Nat Genet.

[35] Hemani G, Zheng J, Elsworth B, Wade KH, Haberland V, Baird D (2018). The MR-Base platform supports systematic causal inference across the human phenome. Elife.

[36] Kamat MA, Blackshaw JA, Young R, Surendran P, Burgess S, Danesh J (2019). PhenoScanner V2: an expanded tool for searching human genotype-phenotype associations. Bioinformatics.

[37] Sintov AC, Yarmolinsky L, Dahan A, Ben-Shabat S (2014). Pharmacological effects of vitamin D and its analogs: recent developments. Drug Discov Today.

